# Screening history and FIGO-stages among Danish women with cervical cancer in 2012–2014: a register-based study

**DOI:** 10.1038/s41598-019-56833-w

**Published:** 2019-12-31

**Authors:** Abir Khalil Bchtawi, Sinem Saritas, Doris Schledermann, René dePont Christensen, Kirsten Marie Jochumsen

**Affiliations:** 1 Faculty of Medicine, University of Southern Denmark, Campusvej 55, 5230 Odense M, Denmark; 2 Faculty of Medicine, University of Southern Denmark, Campusvej 55, 5230 Odense M, Denmark; 3 Department of Pathology, Odense University Hospital, J. B. Winslowsvej 15, 5000 Odense C, Denmark; 4 Research Unit of General Practice, Institute of Public Health, University of Southern Denmark, J.B. Winsløvsvej 9B, 5000 Odense C, Denmark; 5 Department of Gynecology and Obstetrics, Odense University Hospital, Klovervenget 23, 5000 Odense C, Denmark

**Keywords:** Cancer, Risk factors

## Abstract

The objective was to examine whether attendance in the mass cervical screening programme has implications for the prognosis when cervical cancer is diagnosed. We performed a retrospective analysis of all cases of cervical cancer between 1st of January 2012 and 31st of December 2014 in the Region of Southern Denmark. The cases were retrieved from the Danish National Pathology Registry, PatoBank. Odds ratios (OR) with confidence intervals (95% CI) were calculated for attendees versus non-attendees of the screening programme by using χ2-test. 216 patients were included in the study. 61.6% of the study population had not attended the screening programme. Patients who had attended the programme were characterised by disease in low stage (OR = 3.14, 95% CI; 1.66 to 5.92), treatment with surgery alone (OR = 2.63, 95% CI; 1.49 to 4.64) and a lower risk of death (OR = 0.36, 95% CI; 0.15 to 0.87). Adenocarcinomas were more often detected among attendees of the programme compared to squamous cell carcinomas (OR = 4.06, 95% CI; 2.03 to 8.14). Statistically significant results regarding relapse of cancer (OR = 0.62, 95% CI; 0.23 to 1.68, p = 0.47) and lymph node metastases (OR = 0.62, 95% CI; 0.32 to 1.21, p = 0.19) were not found. Cervical cancer detected in women who had attended the mass cervical screening programme prior to the diagnosis, was shown to have a statistically significant lower FIGO stage (p = 0.0004) and was therefore linked to less extensive treatment options. Continued focus on increasing the participation rate of the programme is of importance, as the nonattendance rate continues to be high.

## Introduction

The incidence of cervical cancer has decreased markedly since the onset of mass cervical screening in the 1960s in Denmark. The programme is well-known to detect easily treatable precursors to cancer and thereby reducing the incidence of cervical cancer^1^. However, the incidence of cervical cancer still remains slightly higher in Denmark (14.9/100000 women in 2014, age-standardized) compared to the other Scandinavian countries^2,3^ and the vision of an attendance rate at minimum 75 % countrywide is not yet reached^4^.

In Denmark, women aged 23–49 years are invited for cervical screening every third year by a personally addressed invitation letter and thereafter every fifth year until the age of 65. For women aged 23–59 years, the screening test is a Papanicolaou cytological sample from the uterine cervix, usually taken by the general practitioner. For women aged 60–64 years a high-risk human papillomavirus (hr-HPV) DNA-test is performed on the collected sample. If the hr-HPV DNA-test is negative, the woman is withdrawn from the screening programme, but if the test is positive for hr-HPV 16/18 the woman is referred for colposcopy, and if the test is positive for other hr-HPV types the cytological specimen will be evaluated by microscopy^2^. Participation in the screening programme, follow-up and treatment is free of charge.

The screening programme has continuously been reassessed in several ways to optimise the outcome by reducing the incidence of cervical cancer, but especially in the last 1–2 decades there has been a stagnation of the cervical cancer incidence in Denmark^1,5^. To improve attendance in the cervical screening programme the Danish National Board of Health recommends that women receive two recalls with an interval of 3 months if they do not respond to the first invitation. The general practitioner receives a reminder when follow-up of an abnormal or inadequate cervical sample has failed^2^. Liquid-based cytology has also been introduced in order to reduce the number of inadequate cytological samples and allow for both cytological and HPV test on the same sample^2,6,7^. Although several implementations have been made to enhance the efficiency of mass cervical screening, approximately 400 Danish women are still diagnosed with cervical cancer every year^3^. Three challenges remain predominant: non-attendance to the screening programme, false negative screening samples and lack of follow-up of abnormal or inadequate screening samples. To elucidate how much each of these three reasons contribute to reduce the effectiveness of the programme, the Danish National Board of Health recommended in 2007 and 2012 that new cases of cervical cancer should be reassessed by audit, including re-evaluation of “normal” cervical samples (histological and cytological) up to 3.5 or 5.5 years prior to the cancer diagnosis for the younger and older target populations respectively^2,8^.

The aim of this study was to investigate whether attendance to the mass cervical screening programme is associated with a better prognosis for women diagnosed with cervical cancer focusing on the following prognostic factors: stage of disease, histological diagnosis, lymph node metastases, relapse of cancer and death. It was hypothesised that women attending the screening programme have a better prognosis than non-attendees.

## Methods

### Study design

This register-based study was conducted at Department of Pathology and Department of Gynecology and Obstetrics, at Odense University Hospital, Odense, Denmark. Data were collected retrospectively. The study describes audit results for patients diagnosed with cervical cancer in a three-year study period from 1^st^ of January 2012 to 31^st^ of December 2014 in the Region of Southern Denmark, which has a population of 1.2 million^9^. In that period, 69% of invited women attended the programme^10^.

Medical records concerning information about cancer diagnosis, stage of disease, previous screening history and treatment were examined.

All patients with a histologically verified primary diagnosis of cervical cancer in the study period, living in the Region of Southern Denmark and treated at Odense University Hospital were retrieved from the Danish Pathology Register, PatoBank. The patients were traced in the PatoBank by searching for relevant Systematised Nomenclature of Medicine (SNOMED) codes; T83 for location in the uterine cervix and relevant M-codes for histological cancer diagnoses. The PatoBank is a nationwide database that contains information about all specimens, including cervical samples, examined at departments of pathology in Denmark. The information in the PatoBank was linked to the patients’ electronically medical records by the CPR-number, which is a ten-digit personal identification number assigned to all Danish citizens. The treatment of cervical cancer is centralized to Odense University Hospital in the Region of Southern Denmark.

Information about age and stage of disease (FIGO = International Federation of Gynecology and Obstetrics) at the time of diagnosis as well as information about lymph node metastases (histological and/or on PET-CT), treatment (surgical and/or oncological) and relapse of cervical cancer or death was retrieved from patients’ records. Cytological and histological diagnoses prior to the cervical cancer diagnosis as well as histological diagnosis of the cancer and results from the audit were retrieved from the PatoBank. Follow up was completed in May 2016.

#### Patient and public involvement

Patients or the public were not involved in any way in our work as it was a register based retrospective study.

### The audit process

The Danish National Board of Health provides guidelines for the categorization of the patients into non-attendees (group 1) and attendees (groups 2–7)^1^. In this study we allocated the patients to the same seven groups as listed below. The groups of audit are further elaborated in local guidelines provided for the Department of Pathology at Odense University Hospital, which is also accounted for in the following:

#### Non-attendees

1. **Deficient screening history** (not attended the screening programme) – no cervical samples (cytological or histological) 3.5 years (23–49 years of age) or 5.5 years (50–64 years of age) prior to the diagnosis of cancer.

#### Attendees

2. **Sufficient screening history** (attended regularly in the screening programme without abnormal samples) – at least one “normal” cervical sample test 3.5 or 5.5 years prior to the cancer diagnosis re-evaluated as true negative.
3. **False negative cytology –** a cytological sample defined as “normal” 3.5 or 5.5 years prior to the cancer diagnosis, but diagnosed as High-grade Squamous Intraepithelial Lesion or worse at re-evaluation.
4. **False negative histology** - a histological sample defined as “normal” 3.5 or 5.5 years prior to the cancer diagnosis, but diagnosed as Cervical Intraepithelial Neoplasia grade 2 or worse at re-evaluation.
5. **Lack of follow-up of abnormal or inadequate cytological samples**.
6. **Lack of follow-up of abnormal or inadequate histological samples**.
7. **Other –** includes cases where audit was not feasible due to untraceable samples.

### Preparation techniques

In the Region of Southern Denmark, all the departments of pathology use liquid-based cytology technique (Thinprep® or Surepath®), and three or four departments were using semi-automatic computer assisted screening in the study period.

### Statistical analyses

A χ^2^-test was performed on the groups 2–7 versus group 1 using SPSS version 23. Only results with p-values <0.05 were considered statistically significant.

## Results

**Figure 1:**
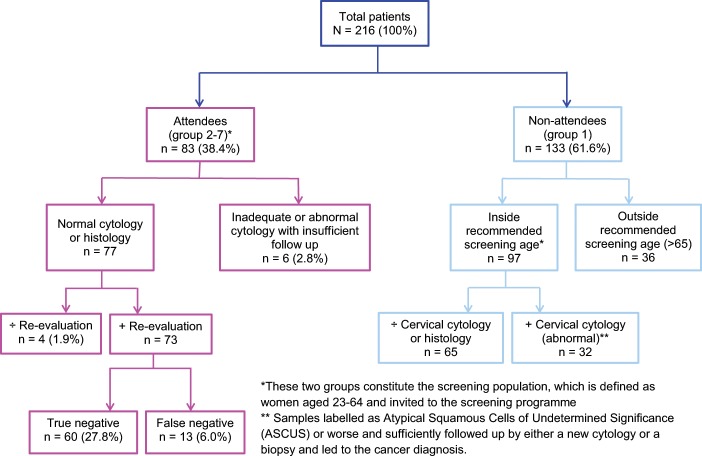
Flowchart showing the distribution of the patients in the 7 audit groups.

A total of 216 patients diagnosed with cervical cancer were found to fulfil the inclusion criteria for this study. The mean age of the patients was 48.5 ± 16.8 (range 23–95). The study results are shown in Table 1, and an overview of the distribution of the study population between the audit groups 1–7 is seen in Fig. 1.

**Table 1:** Results of the Danish screening study and statistical analyses.

	Total cancers *N* = *216 (100 %)*	Group 1 *N* = *133 (61.6 %)*	Group 2 *N* = *60 (27.8 %)*	Group 3 *N* = *13 (6 %)*	Group 4 *N* = *0 (0 %)*	Group 5 *N* = *6 (2,8 %)*	Group 6 *N* = *0 (0 %)*	Group 7 *N* = *4v(1.9 %)*	Statistic Group 2–7 vs. Group 1
	n (%)	n (%)	n (%)	n (%)	n (%)	n (%)	n (%)	n (%)	OR (95 % CI) *p-value*
**Mean age (range)**	48.5 (23–95)	52.4 (23–95)	42,9 (24–81)	43,2 (30–60)	—	36.3 (25–67)	—	38,3 (27–60)	
**Age bands (years)**									
23–49	132 (61.1)	70 (52.6)	44 (73.3)	10 (76.9)	—	5 (83.3)	—	3 (75.0)	
50–64	43 (19.9)	27 (20.3)	12 (20.0)	3 (23.1)	—	—	—	1 (25.0)	
>65	41 (19.0)	36 (27.1)	4 (6,7)	—	—	1 (16.7)	—	—	
**Histological diagnosis**									4.06 (2.03 to 8.14)^α^
SCC^a^	162 (75.0)	112 (84.2)	36 (60.0)	7 (53.8)	—	5 (83.3)	—	2 (50.0)	***< 0.0001***
AC^b^	45 (20.8)	16 (12.0)	22 (36.7)	5 (38.5)	—	—	—	2 (50.0)	
ASC^c^	9 (4.2)	5 (3.8)	2 (3.3)	1 (7.7)	—	1 (16.7)	—	—	
**FIGO stage**									3.14 (1.66 to 5.92)^β^
I	140 (64.8)	73 (54.9)	47 (78.3)	11 (84.6)	—	5 (83.3)	—	4 (100.0)	***0.0004***
II	48 (22.2)	36 (27.1)	10 (16.7)	1 (7.7)	—	1 (16.7)	—	—	
III	17 (7.9)	14 (10.5)	2 (3.3)	1 (7.7)	—	—	—	—	
IV	10 (4.6)	9 (6.8)	1 (1.7)	—	—	—	—	—	
Unknown	1 (0.5)	1 (0.8)	—	—	—	—	—	—	
**Treatment**									2.63 (1.49 to 4.64)^γ^
Surgery alone Other treatments:	106 (49.1)	53 (39.8)	37 (61.7)	9 (69.2)	—	3 (50.0)	—	4 (100.0)	***0.0008***
- Surgery and adjuvant	24 (11.1)	14 (10.5)	7 (13.3)	1 (7.7)	—	1 (16.7)	—		
oncological therapy								—	
- Curative intended oncological therapy	61 (28.2)	45 (33.8)	13 (21.7)	2 (15.4)	—	1 (16.7)	—		
- Palliative oncological therapy	14 (6.5)	14 (10.5)	—	—	—	—	—		
Individual therapy^*^	10 (4.6)	6 (4.5)	2 (3.3)	1 (7.7)	—	1 (16.7)	—	—	
- No therapy^**^	1 (0.5)	1 (0.8)	—	—	—	—	—	—	
**Lymph node metastasis**									0.62 (0.32 to 1.21)^δ^
Yes	56 (25.9)	39 (29.3)	15 (25.0)	2 (15.4)	—	—	—	—	***0.19***
No	114 (56.9)	72 (54.1)	35 (58.3)	10 (76.9)	—	5 (83.3)	—	1 (25.0)	
Unknown^***^	37 (17.1)	22 (16.5)	10 (16.7)	1 (7.7)	—	1 (16.7)	—	3 (75.0)	
**Relapse of cancer**									0.62 (0.23 to 1.68)^π^
Yes	19 (8.8)	13 (9.8)	4 (6.7)	2 (15.4)	—	—	—	—	***0.47***
No	176 (81.5)	102 (76.7)	54 (90.0)	11 (84.6)	—	5 (83.3)	—	4 (100.0)	
Not relevant^****^	17 (7.9)	15 (11.3)	1 (1.7)	—	—	1 (16.7)	—	—	
Unknown^*****^	4 (1.9)	3 (2.3)	1 (1.7)	—	—	—	—	—	
**Death**									0.36 (0.15 to 0.87)^η^
Yes	34 (15.7)	27 (20.3)	5 (8.3)	1 (7.7)	—	1 (16.7)	—	—	***0.02***
No	182 (84.3)	106 (79.7)	55 (91.7)	12 (92.3)	—	5 (83.3)	—	4 (100.0)	

^a^Squamous cell carcinoma.

^b^Adenocarcinoma.

^c^Adenosquamous carcinoma.

*Includes patients who received surgical and/or oncological therapy beyond common guidelines and who could not be categorized into the groups of treatment.

**One patient in the study abstained from treatment. This patient was excluded from the statistical analysis regarding treatment.

***Includes patients with no lymph node removement and no PET-CT executed and thus status on lymph node metastases was unknown. These patients were excluded from the statistical analysis regarding lymph node metastases.

****Includes patients who were never declared healthy. These patients were excluded from the statistical analysis regarding relapse of cancer.

*****Includes patients with no medical records regarding relapse of cancer. These patients were excluded from the statistical analysis regarding relapse of cancer.

^α^Statistical analysis on AC versus SCC among the groups 2–7 versus group 1.

^β^Statistical analysis on FIGO stage I versus FIGO stage II and higher among the groups 2–7 versus group 1.

^γ^Statistical analysis on surgery versus the other treatments listed above among the groups 2–7 versus group 1.

^δ^Statistical analysis on lymph node metastases among the groups 2–7 versus group 1.

^π^Statistical analysis on relapse of cancer among the groups 2–7 versus group 1.

^η^Statistical analysis on death among the groups 2–7 versus group 1.

We found that 133 (61.6%) of 216 patients had a deficient screening history, of whom 36 (27.1%) were outside (older than) the recommended screening age and 97 (72.9%) within the recommended screening age. Thus, 61.6% of the study population were non-attendees of the screening programme, Fig. 1. The distribution of attendees among the audit groups 2–7 is shown in Table 1.

Re-evaluation of cytological samples revealed false negative cervical samples in 13 (6.0%) patients. In six (2.8%) cases, follow-up of cervical cytological samples had failed and in four cases (1.9%) audit was not feasible due to untraceable cytological glass slides.

**Figure 2:**
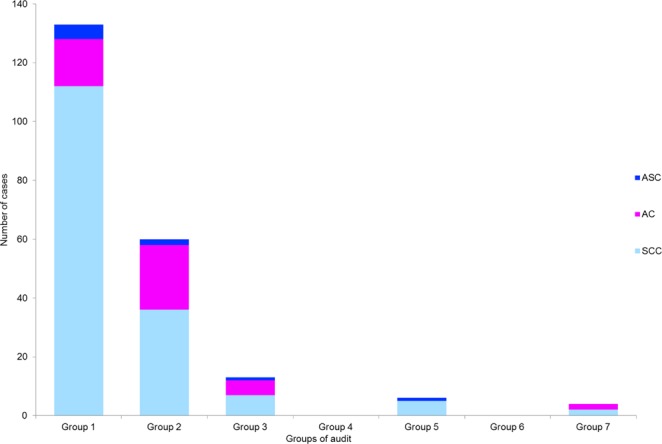
Distribution of histologic subtypes in each cervical cancer audit group.

The distribution of histological subtypes in the seven audit groups is shown in Fig. 2. Adenocarcinomas were overrepresented among attendees (34.9%) compared to non-attendees (12.0%). The risk of being diagnosed with adenocarcinoma compared to squamous cell carcinoma was higher among attendees compared to non-attendees of the screening programme (OR = 4.06, 95% CI; 2.03 to 8.14, p < 0.0001). Neuroendocrine carcinomas and other rare histological diagnoses were not found in this study.

Among the 83 patients who had attended the screening programme, a total of 67 (80.7%) were diagnosed with FIGO stage I^11^ compared to 73 (54.9%) of the 133 patients who had not attended the programme, meaning that 19.3% of the attendees and 45.1% of the non-attendees were diagnosed with FIGO stage II and higher. For one patient the stage of disease was unknown as she abstained from further investigation and was consequently excluded from the statistical analysis regarding FIGO stage. Attendance to the screening programme was associated with higher odds for being diagnosed with less advanced stage of disease (FIGO stage I) compared to non-attendance (OR = 3.14, 95% CI; 1.66 to 5.92 p = 0.0004).

A total of 106 (49.1%) patients received surgical treatment alone, while 109 (50.5%) patients received other treatment, of whom 14 (6.5%) received palliative oncological therapy alone because of FIGO stage IV and/or comorbidity; all 14 patients were non-attendees, Table 1. One patient abstained from therapy and was excluded from the statistical analysis regarding treatment. Among the 83 attendees of the screening programme, 53 (63.9%) received surgical treatment alone compared to 53 (39.8%) of the 133 non-attendees. Our data revealed a statistically significant association between attending the screening programme and receiving surgical treatment alone compared to other treatments, when diagnosed with cervical cancer (OR = 2.63, 95% CI; 1.49 to 4.64, p = 0.0008).

Of the 133 non-attendees of the screening programme, 39 (29.3%) patients had lymph node metastases at the time of diagnosis, while 17 (20.5%) of the 83 attendees of the programme had lymph node metastases. Additionally, 13 (9.8%) patients among the non-attendees and six (7.2%) among the attendees had relapse of cancer. Statistically significant results regarding lymph node metastases (OR= 0.62, 95% CI; 0.32 to 1.21, p = 0.19) and relapse of cancer (OR= 0.62, 95% CI; 0.23 to 1.68, p = 0.47) were not achieved in this study.

In our study, a total of 34 (15.7%) patients were registered dead (unknown cause of death); 27 (20.3%) among non-attendees and 7 (8.4%) among attendees. Attendees of the screening programme had a lower risk of death (OR = 0.36, 95% CI; 0.15 to 0.87, p = 0.02) compared to non-attendees.

## Discussion

We investigated whether attending a well-organized screening programme^5^ affects the prognosis of cervical cancer. The odds for being diagnosed with a less advanced disease stage (FIGO stage I) were 3.14 times higher given that attendance to the screening programme had been adequate. This is consistent with previous studies, which emphasize a higher risk of advanced stage of disease when not attending a screening programme^12,13^. Similarly, attendees of the screening programme had 2.63 times higher odds for being sufficiently treated by surgery alone and not requiring more extensive treatment options. This was an expected observation, as FIGO stage I (80.7%) dominated among attendees of the screening programme. Furthermore, the risk of death was found to be lower among attendees of the screening programme. However, cause of death was unknown and follow-up of the patients varied. Among the 133 non-attendees, 36 women were outside the recommended screening age (older), and consequently their risk of death of any cause is higher. In the study of Kristensson *et al*.^14^, non-attendees were found to be socio-economically disadvantaged compared to attendees and several other studies outlines socioeconomic factors as important variables for screening behaviour and also stage of cervical cancer^14–16^. Thus, higher age and poor socioeconomic factors among the non-attendees could also have influenced the results regarding death, but this was not further investigated.

We found a trend towards a lower risk of lymph node metastases and relapse for women who attended the screening program, but data were not statistically significant (p = 0.19 and p = 0.47 respectively). Furthermore, it was difficult to extract data regarding lymph node metastases, as this information relied on histology for some patients, while PET-CT was the determinant for other patients. For the latter, information about lymph node metastases was not always consistently reported. It is also important to underline that follow-up of the study population regarding relapse of cancer has varied as a result of a divergent time of cancer diagnosis. Although attending the screening programme seems to improve the prognosis of cervical cancer, a larger sample is required to investigate the same positive effect of attendance on lymph node metastases and relapse of cancer.

61.6% of the women with cervical cancer had not attended the screening programme, i.e. the women either did not respond to the invitation letter or to the two recalls and for some cases or the women did not fulfil the criteria of being an attendee (the women were actually older than the recommended screening age). Non-attendance rates between 54–64% have previously been reported^12,17,18^, which is consistent with the rate found in this study. A recent Danish study from the Capital Region of Denmark reports a somewhat lower non-attendance rate of 45.5%^19^. This difference may be explained by different demographics of the two study populations, as Kirschner *et al*^19^. describes an urban population of women, while our study describes a population from both urban and rural areas. This may reflect diversity in the screening behaviour between the two study populations. However, when reporting a non-attendance rate, women outside the recommended screening age should not be taken into account. The non-attendance rate reflects the efficiency of the screening programme and should thus be preserved women within the screening population but with absence of a cervical cytology available for re-evaluation. It seems of continued relevance to place emphasis on increasing the participation in the cervical screening programme as attendees of the screening programme in this study trended to have less advanced stage of disease at time of diagnosis. HPV self-testing for women who do not respond to the two recalls could be one option to detect this major known risk factor for cervical cancer development^20–22^. In the recent guidelines from The Danish National Board of Health from 2018, it is recommended to offer the possibility of self-sampling in the up-coming years, to enhance the participation in the screening programme^23^. This option is partially implemented in some regions in Denmark.

Of the patients included, 6.0% had false negative cytology, which is comparable to the 9.8% reported by Kirschner *et al*.^19^, which reflects an overall proper reading of cytological glass slides. However false negative cytology results from interpretation or location bias and continued improvement of both collection and interpretation techniques is required.

In this study, 27.8% of the patients with cervical cancer had attended the screening programme and were diagnosed with malignant disease despite all previous samples were judged normal; in other words they had developed interval cancers. It has been debated whether interval cancers are due to sampling errors or are an expression of rapidly developing cancers^13,18,19^. Interval cancers reflect incompleteness in the screening programme. Thus, the programme cannot be an isolated preventive approach against cervical cancer of which the HPV vaccine has been an additional approach since 2006^24^. In 2009, the HPV vaccine became a part of the Danish childhood vaccination programme recommending HPV vaccination of girls at the age of 12^25^. Furthermore the 9-valent HPV vaccine (Gardasil 9) is now also offered to boys turning 12 years in summer of 2019, thereby enhancing heard immunity^26^. It is expected that the dual impact of HPV vaccination for both girls and boys, and the cervical screening programme will further reduce the incidence of cervical cancer.

Several studies highlight that it is more difficult to detect adenocarcinoma compared to squamous cell carcinoma through the screening programme^27–30^. This is partly explained by the risk of sampling errors, as endocervical components are more difficult to collect and can be difficult to identify and diagnose by cytological examination^31,32^. Adenocarcinomas were more often found among attendees compared to non-attendees in this study, which may reflect deficiency in the screening process, as pre-cursors to adenocarcinomas were not detected prior to the cancer diagnosis. In addition, adenocarcinomas were detected in 22 (36.7%) of the 60 patients with true negative cytology, i.e. group 2, and in five (38.5%) of the 13 patients with false negative cytology, i.e. group 3. These findings emphasize the importance of sampling techniques and the need for continued attention on the interpretation of cytomorphological changes in cells of glandular origin. Implementation of HPV-DNA tests as the primary screening method might lead to improved detection of glandular lesions. The Danish National Board of Health has recently requested the regions to conduct a controlled, differentiated implementation of HPV screening for the age-group 30–59 years of age which would allow for an evaluation of the efficiency of HPV screening versus cytological sampling^23^.

Of the 216 patients who had developed cervical cancer, 41 (19.0%) were above 65 years of age and thus outside the recommended screening age. Only one of these patients had a history with conization because of cervical dysplasia prior to the diagnosis of cervical cancer. Hammer *et al*. proposes to extend the screening population to include women above 65 years of age, as the incidence of cervical cancer among elderly women remains high^33^. However, according to Lynge *et al*. the current high incidence of cervical cancer among elderly women may represent residuals of unscreened or underscreened birth cohorts^34^. Further decrease in the incidence of cervical cancer among the elderly women is expected, as the present recommendations offer women aged 60–64 years a hr-HPV DNA-test exiting the screening programme with follow-up of the hr-HPV positive women. Thus, it is debatable whether an expansion of the screening population will be more beneficial than the present actions. Future results upon the incidence of cervical cancer among the elderly women, taking the current guidelines into account, are required to determine the efficiency of the hr-HPV DNA-test and decide whether further initiatives are necessary.

This study was strengthened by the use of PatoBank, which allowed access to all available cervical samples for all relevant cervical cancer cases in Denmark. Furthermore, the unique CPR-system allowed for accurate linkage of information between the PatoBank and medical records. All cases of cervical cancer detected in 2012–2014 in the Region of Southern Denmark were included and the diagnoses of cervical cancer were further confirmed, as Systematised Nomenclature of Medicine codes were compared to information in medical records. A limitation in this study was the limited time of follow-up and the lack of information about the cause of death. Furthermore, baseline characteristic linked to screening behaviour as socioeconomic factors was not feasible and thus not adjusted for in the analysis.

During this study, we gained insight into the performance of the audit process in the Region of Southern Denmark, and as we experienced several challenges connected to this procedure, it is of relevance to comment on these. The audit process intends to ensure the quality of the detection and treatment of cervical cancer^2^. Conclusively, the patients are either classified as attendees or non-attendees of the screening programme. Hence, the audit process can be regarded as a quality assurance of the screening programme, which is an opinion appearing explicitly in the audit guidelines in the United Kingdom^35^. Thus, in the audit process, it only seems sensible to re-evaluate true screening tests, i.e. cervical samples taken upon an invitation to the screening programme. However, all cervical samples coded as “normal” in the Danish PatoBank up to 5.5 years prior to the cancer diagnosis are being re-evaluated, without distinguishing between true screening tests and tests taken outside the screening programme, e.g. tests taken on medical indication or opportunistic tests. The reason for that is not further explained, but it can be assumed that it is being done for legal reasons (compensation claims). All women with “normal” cervical samples up to 5.5 years from the cancer diagnosis are, in the audit process, considered as attendees of the screening programme and the word “attendee” is consequently misused. Ideally, it should be preserved to women actually attending the screening programme upon an invitation, but the current coding practice does not make this possible. It is difficult to conclude from the information in PatoBank whether the cervical cancer actually was detected upon screening (actual attendee) or on symptoms, as symptoms may have occurred in a screening interval period. A woman could possibly attend her general practitioner with contact-bleeding (symptom) and be an “attendee of the mass cervical screening program” if a specimen was found in her PatoBank history up to 5.5 years prior to cancer diagnosis. If the purpose of an audit is to assure the quality and efficiency of the screening programme, it could be considered to re-organize the present audit process, so only true screening tests influence the audit conclusions. Re-organization of the audit process could involve attachment of more precise information to the samples in the PatoBank, which would make it easier to distinguish between true screening tests and tests taken outside the screening programme. Inspiration can be drawn from the United Kingdom, where the audit process is able to differentiate between women who had attended the screening programme and women who had not when developing cervical cancer, especially with focus on the reasons for non-attendance in spite of an invitation^36^.

## Conclusion

A trend of a lower stage of disease at diagnosis, less extensive treatment of cervical cancer and lower risk of death was found among attendees of the screening programme. Besides lowering the incidence, attendance was shown to improve prognosis for women who are diagnosed with cancer despite regular cervical cytology tests. More focus on the screening programme and its ability to detect adenocarcinomas is also desirable, as the proportion of adenocarcinomas among the attendees of the screening programme with true negative (36.7%) and false negative cytology (38.5%) appears rather high.

Our study results thus provide a mapping of screening behaviour and prognostic factors among women diagnosed with cervical cancer and thereby add a useful baseline for additional studies with nationwide data reviewing the efficiency of the screening programme. Further clinical information following cervical samples could add to the audit process’ ability to differentiate between women who had attended the screening programme and women who had not when developing cervical cancer. An optimization of the audit process as well as national data covering the screening behaviour in Denmark are needed to clarify the complete efficiency of the screening programme.

### Ethical approval and consent to participate

The study was approved by the Danish Data Protection Agency (reference no. 16/4807, date: 03/02-16) and the Danish Patient Safety Authority (file no. 3-3013-1520/1/, date: 31/03-16). These authorities determined that informed consent was not required because the study used de-identified data from approved databases.

## Data Availability

The datasets used and/or analysed during the current study are available from the corresponding author on reasonable request.
